# Peripheral Inflammatory Biomarkers of Methamphetamine Withdrawal Patients Based on the Neuro-Inflammation Hypothesis: The Possible Improvement Effect of Exercise

**DOI:** 10.3389/fpsyt.2021.795073

**Published:** 2021-12-23

**Authors:** Jingsong Wang, Chunxia Lu, Lan Zheng, Jun Zhang

**Affiliations:** ^1^Key Laboratory of Physical Fitness and Exercise Rehabilitation of Hunan Province, Hunan Normal University, Changsha, China; ^2^Hunan Judicial Police Vocational College, Changsha, China

**Keywords:** aerobic combined resistance training, IL-1β, TNF-α, IL-6, craving degree

## Abstract

Methamphetamine (MA) induced addiction and neuroinflammation has been implicated. Based on the neuroinflammation hypothesis, this study aims to investigate how exercise influences the craving of patients in MA withdrawal, and explore the mechanism of peripheral inflammation. A total of 90 patients in MA withdrawal were recruited. No difference was noted in the number of years of drug use and the frequency of drug use among patients, and the withdrawal time was within 2 months. The subjects were grouped based on the degree of craving induced by the cues: non-craving control group (NCC group), craving control group (CC group), and craving exercise group (CE group). The CE group was subjected to aerobic combined resistance training. Then, the ELISA method was used to detect plasma IL-6, TNF-α, and IL-1β concentrations; Visual Analog Scale (VAS) measurement of cue-induced cravings under Virtual Reality (VR) exposure (VR-VAS) and the Desires for Drug Questionnaire (DDQ) were used to assess cravings. Consequently, plasma IL-6, TNF-α, IL-1β, levels, and the VR-VAS and DDQ scores of MA withdrawal patients were significantly reduced after exercise. This study confirmed that 8 weeks of incremental load aerobic combined with resistance training reduces peripheral inflammation and significantly reduces the level of craving for MA.

## Introduction

Methamphetamine (MA) is an addictive drug, and its abuse of MA causes irreversible structural damage to the brain regions involved in regulating cognitive functions (1). The existing literature shows that the abuse of psychostimulant drugs triggers certain damage to the blood-brain barrier (BBB) and neurotoxicity (2). Exposure of animals to MA induced neurotoxicity through several molecular and cellular mechanisms, including various receptors, energy metabolism, inflammation, and immune system activation (3).

The neuroinflammation hypothesis states that MA influences the activity of glial cells, which in turn triggers addictive behavior and neurotoxicity (2, 4). Microglia are immune cells in the brain; when activated, they easily cause neuronal damage since they secrete chemokines and cytokines. On the other hand, astrocytes are abundant in the central nervous system (CNS), which protects the brain and maintains brain homeostasis (3). At the same time, astrocytes secrete various cytokines, including tumor necrosis factor, interleukins, and chemokines that activate MA-induced neurotoxicity (5). One study found that activation and proliferation of microglia in the CNS among patients in MA withdrawal showed a proliferation trend for at least 2 years (6).

Notably, MA disrupts the peripheral and central immune functions. At the same time, inflammatory cytokines produced by these changes enter the central nervous system of the brain where they induce glial cells to produce similar inflammatory cytokines, thereby providing a highly inflammatory environment for central immunity (7). Patients in MA withdrawal seeking treatment suffer from severe neuropsychiatric complications (cognitive impairment, etc.), which persist after discontinuation of use negatively affecting recovery outcomes (8, 9). A study in mice treated with MA showed that peripheral and central nervous system immune disorders and cytokines (IL-6, TNF-α, and IL-β) revealed specific changes in peripheral plasma and brain regions (9). Besides their roles in rewarding effects of MA, changes in immune function promote the development and persistence of cognitive deficits in chronic human MA abusers. For instance, increased plasma levels of pro-inflammatory cytokines IL-1β, IL-2, IL-6, and TNF-α and chemokines MCP-1, MIP-1α, and MIP-1β have been significantly associated with greater neurocognitive dysfunction among human MA users (10). Reports indicate that adults recovering from early MA dependence exhibit peripheral immune deficiency, and changes in immune cytokines activated by MA increase with increasing cognitive impairment (10, 11). Since MA can induce an increase of IL-1β, TNF-α, IL-6 in peripheral blood plasma and addiction-related brain function areas (brain hippocampus, striatum, etc.), as well changes are more apparent; at the same time in withdrawal, the period continues to rise and can be maintained for at least 2 years (11, 12). Thus, peripheral blood plasma IL-1β, TNF-α, IL-6 were selected to confirm whether it can be used as a peripheral immune marker for addiction among patients in MA withdrawal.

Cue-induced cravings are part of addiction and easily cause relapse (12, 13). Under laboratory conditions, the measurement of cue-induced cravings under VR exposure indicates the severity of addiction (14, 15). Virtual reality (VR) is a group of seemingly real virtual worlds comprising images and sounds. It is a computer-generated simulation of the environment aimed at immersing the user in the environment. It is delivered to users via visual and auditory senses, making them feel immersive (16). The immersive nature of VR is used to modulate various sensory stimuli, evaluate and induce related pathological behaviors and feelings (for example, cravings), as well as possible behavioral responses (17). Besides, it helps the subjects learn how to better address their problems. Park et al. (2019) reported that besides addictive psychopathology, VR can be used for other mental illnesses (18). Therefore, the VR equipment is extensively used in the field of medical treatment and psychological treatment of China's judicial administration drug treatment system; it is specifically used to diagnose or evaluate the degree of craving, isolate the interference of internal and external environmental factors to a certain extent, and create real experience and feelings for patients.

Other studies indicate that moderate-intensity aerobic exercise reduces craving in MA- withdrawal patients individuals (9, 19, 20). At the same time, aerobic combined resistance training significantly reduces anxiety and depression associated with MA withdrawal patients; also, it improves aerobic capacity, muscle endurance, and strength, heart rate variability, as well as promote recovery from drug dependence (20–24). Additionally, long-term moderate-intensity exercise reduces inflammation levels (25). Studies show that exercise does not induce sufficient clinical benefits (26). Although exercise relieves depression symptoms and improves physical fitness, it cannot reduce drug consumption (26). Therefore, this calls for additional investigations into the mechanisms and methods. Based on the hypothesis of neuroinflammation, the level of inflammation in the peripheral blood of patients with MA withdrawal can be used as a plasma inflammatory marker to induce craving under VR exposure. Exercise reduces MA-related pathologies hence showing therapeutic effects. Therefore, we evaluate whether aerobic combined resistance training can reduce the cue-induced cravings of MA abstinence individuals, and explore its peripheral inflammatory mechanisms. This is geared toward providing a theoretical reference for reducing drug cravings among patients in withdrawal and providing protection to individuals withdrawing from drugs.

## Subjects and Methods

### Subjects

**Figure 1:**
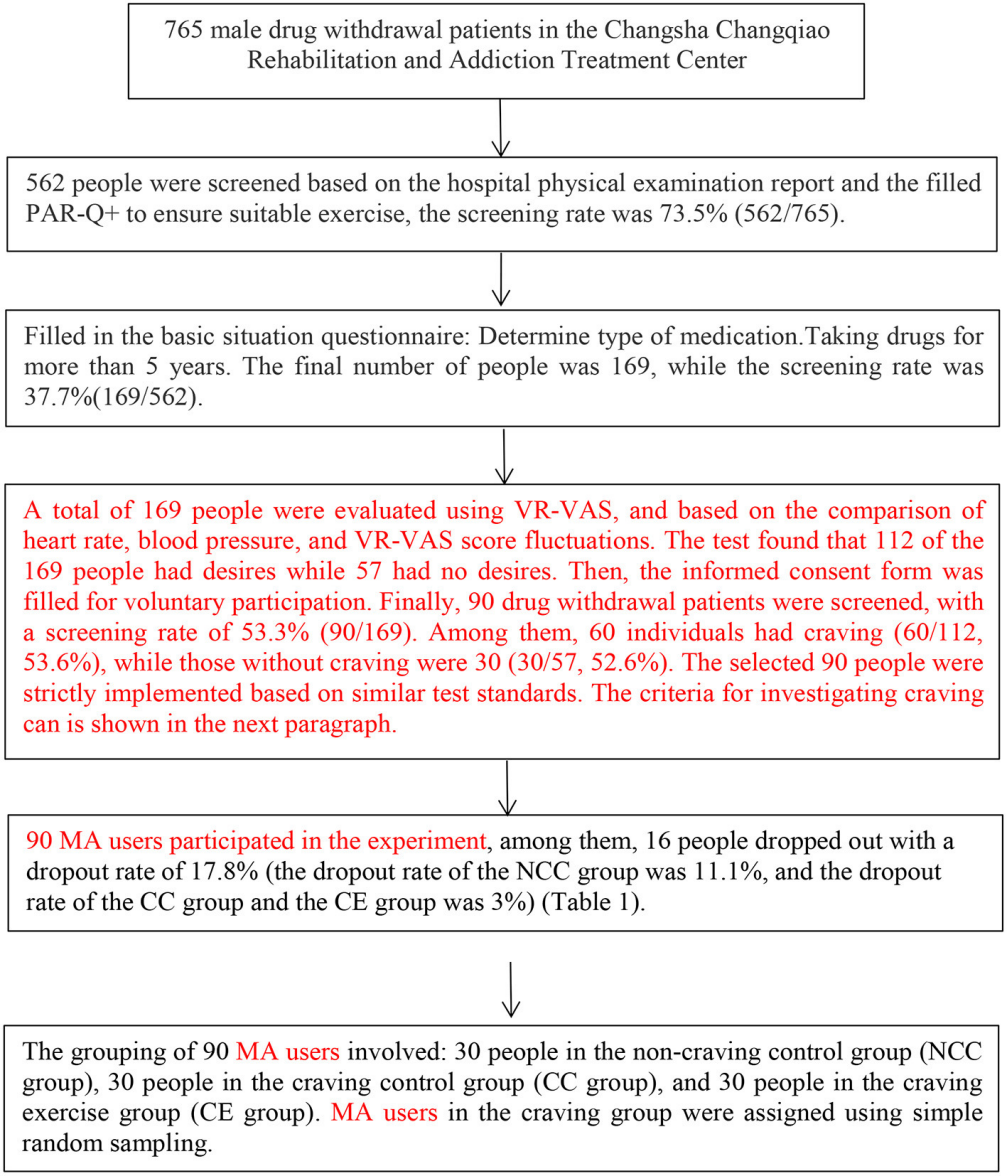
Screening flow chart.

This study recruited 765 male MA users undergoing supervised withdrawal from the Changsha Changqiao Rehabilitation and Addiction Treatment Center in Hunan Province. The selection criteria included: (1) All participants were male patients in MA withdrawal and combination with other drugs; (2) More than 2 years of drug use; (3) Snorting as the means of taking drugs; (4) No other diseases including systemic disease, metabolic diseases, autoimmune diseases, cerebrovascular diseases, and family genetic diseases; (5) No anti-inflammatory drugs and β-blockers taken within 1 month before and during the intervention; (6) All the participants filled in a Physical Activity Readiness Questionnaire+(PAR-Q+) to ensure their suitability for exercise. The investigation process is shown in Figure 1.

This study was approved by the Ethics Committee of Hunan Normal University (Batch number:16-2010) and was conducted according to the ethical requirements of clinical trials. All subjects signed the informed consent. The dropout rate of 16 dropouts was 17.8% (the dropout rate for the NCC group was 11.1%, while that for both the CC group and the CE group was 3%). The remaining 74 were included in the study, including the NCC group (20 people), CC group (27 people), CE group (27 people). The criteria for dividing the degree of craving was abstinence with craving had increased heart rate and blood pressure during VR-VAS (*p* < 0.05) with a score range of 11–100. This method can eliminate the possibility of subjectively selecting samples. Alternatively, abstinence with decreased heart rate and constant blood pressure were assigned to a group of patients (*p*> 0.05) with a score interval between 0 and 10 (27–32) (Table 1). All the cravings of the three groups were measured under a similar cue-induced craving situation; notably, the NCC group was not sensitive to cue-induced cravings.

**Table 1:** Basic information.

**Content**	**NCC group** **(***n =*** 20)**	**CC group** **(***n =*** 27)**	**CE group** **(***n =*** 27)**
Age(year)	37.15 ± 9.71	33.55 ± 6.53	36.75 ± 9.30
Height(cm)	166.0 ± 5.59	166.4 ± 5.78	167.95 ± 4.45
Weight(kg)	66.50 ± 8.96	61.45 ± 8.01	65.95 ± 7.32
History(month)	74.55 ± 8.00	73.20 ± 12.32	72.25 ± 10.36
The length of withdrawal patients (month)	2.95 ± 2.06	3.00 ± 1.90	3.59 ± 2.59
Frequency (week)	8.45 ± 3.83	10.40 ± 5.15	12.80 ± 8.56
Intensity per time (g)	0.47 ± 0.27	0.36 ± 0.30	0.63 ± 0.48
VR-VAS(score)	5.65 ± 2.94	59.63 ± 16.92	59.59 ± 22.67

### Methods

#### Exercise Intervention

**Figure 2:**
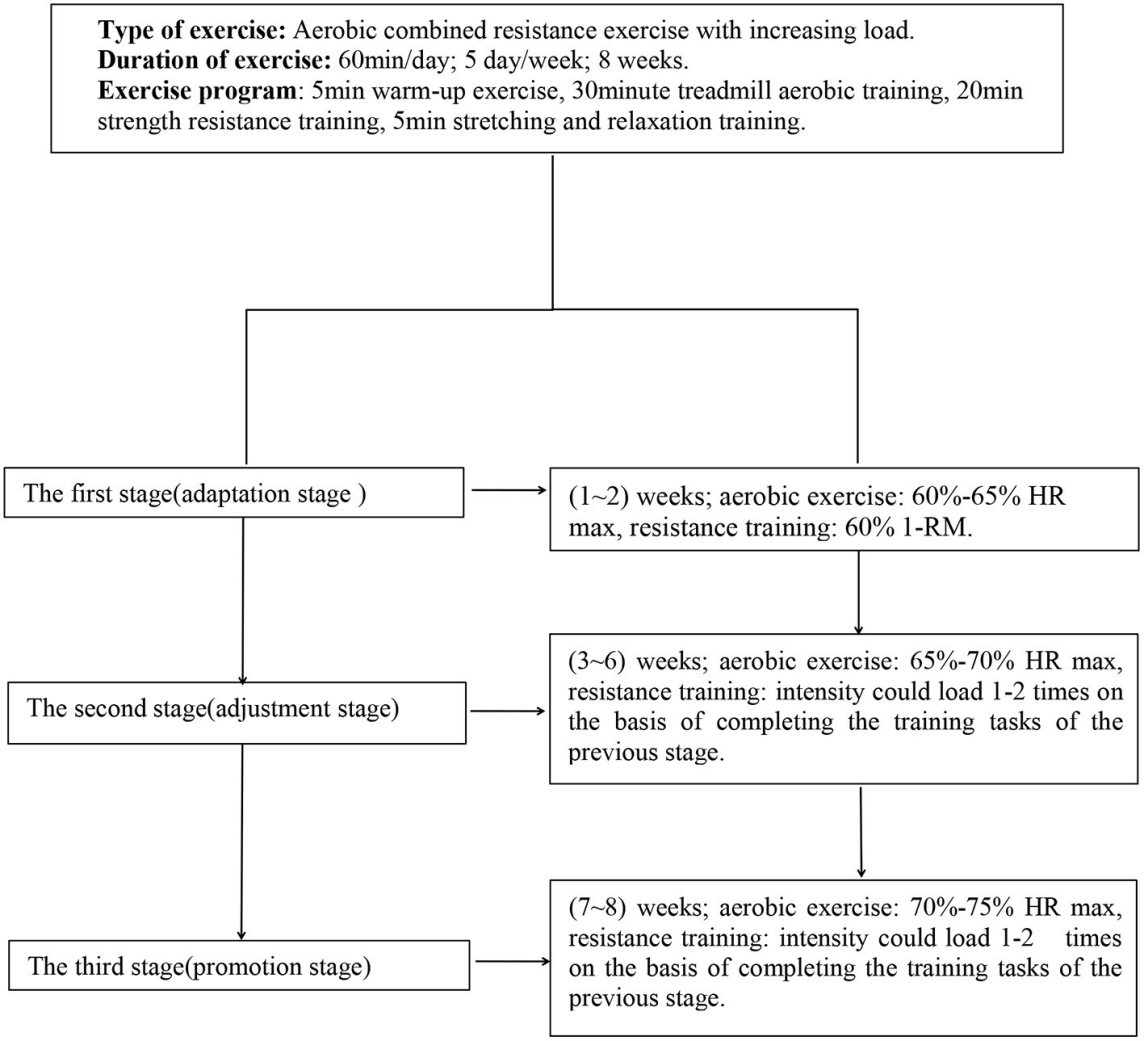
Exercise intervention flow chart.

Aerobic combined resistance exercise is considered a good adjuvant treatment for MA withdrawal patients (20–24). This exercise intervention program follows ACSM's recommendations (33). Here, 27 people in the exercise group were subjected to structured and progressive exercise intervention, while the control group received safety and health education. The maximum heart rate (HRmax = 206.9–0.67 × age) and maximum weight (1RM) of each individual were determined before exercise for one repetition. The specific content of the exercise intervention program was as follows: Each practice time was 60 min, five times a week, practice 8 weeks. The exercises for the muscle groups were divided into the upper limb and lower limb muscle groups, strength resistance training was aimed at improving muscle strength and explosive power, with 8–12 times for each group, two sets for each device, 60 s intervals for each set (Figure 2).

### PAR-Q+(Physical Activity Readiness Questionnaire+)

PAR-Q+ is an internationally recognized health risk assessment questionnaire. Exercise prescribers or qualified coaches use it to understand the health history, symptoms, and risk factors of subjects, and possible risks and safety during exercise. At the same time, individualized, safe, and effective exercise prescriptions are designed for subjects based on the evaluation results. PAR-Q+ includes seven general health problems and 10 supplementary problems for diseases. If the answer is “no”, the subject is not allowed to participate in physical activities, which follows the general physical activity guidelines for healthy asymptomatic people (34). Of note, the participant is only cleared for physical activity if he or she answers no to all of the follow-up questions on pages two and three. Our survey showed that 203 (26.5%) individuals were unsuitable for exercise, whereas 562 (73.5%) people were suitable.

### Measurement of Craving

#### Desires for Drug Questionnaire (DDQ)

DDQ was originally used for self-evaluation of instant cravings for heroin withdrawal patients. Nonetheless, recent studies have also applied it to investigate the short-term craving process of MA withdrawal patients in clinical trials (35). The questionnaire comprises three dimensions: desire and intention (the average of the sum of questions 1, 2, 4, 6, 9, 12, and 13; desire and intention: desire for MA); negative reinforcement (the average of the sum of questions 5, 8, 10, and 11; negative reinforcement: repeated use of MA to relieve withdrawal symptoms), and control (the average of the sum of questions 3 and 7, control: the ability to control MA). DDQ and VAS jointly measure the craving degree with a high correlation. DDQ sub-scales provide specific information complementing the global quantitative assessment from VAS.

#### Cue-Induced Craving During VR Exposure (VR-VAS)

VAS was previously used for heroin. This study used a modified version for evaluation (36), which included a 100 mm visual analog scale (VAS) and adjective modifiers (27–32) (“0–10: no, 11–30: mild, 31–60: moderate, 61–100: extreme”).

**Figure 3:**
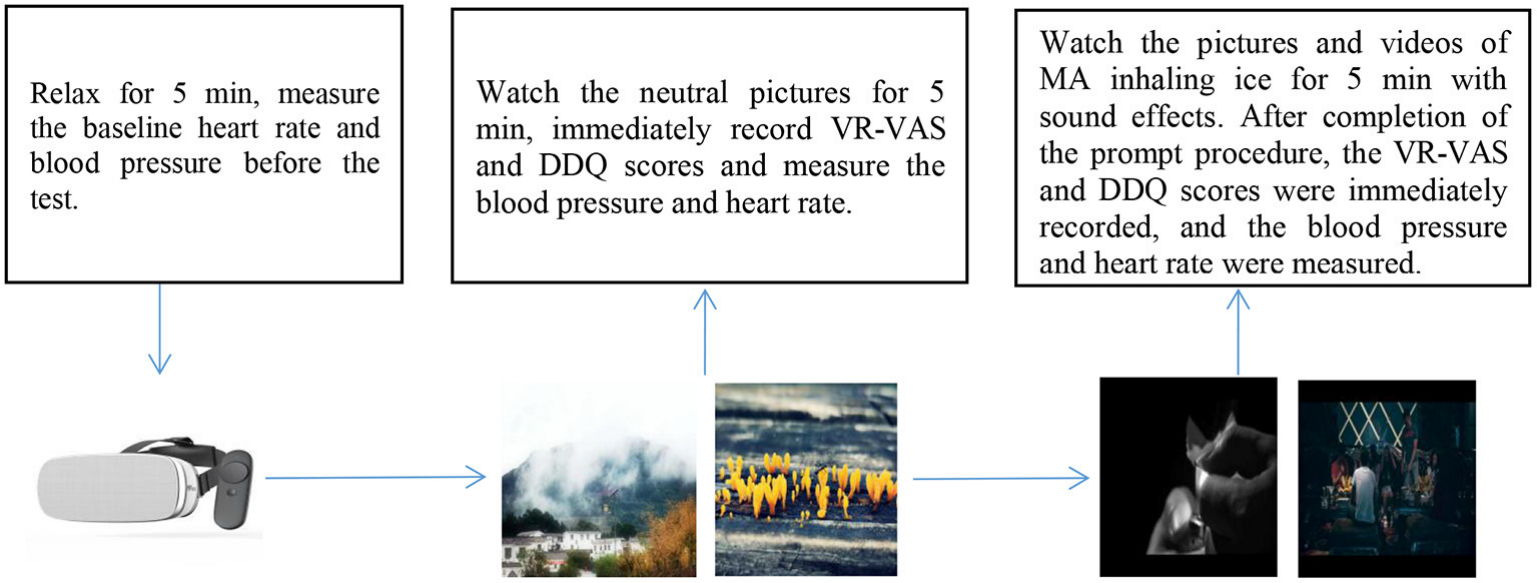
Cue-induced craving test flow chart.

In this study, head-mounted VR devices (Pico Goblin A7215), headsets, and a 0–100 mm VAS scale (0 means “no craving”, while 100 means “extreme craving”) were used to trigger a craving for MA cues during VR exposure. Then the DDQ score was immediately determined after the evaluation. The VR-VAS and DDQ assessments were performed on the same day as the blood draw, and three individuals were assessed each time. The test procedure involved: The participants were required to relax for 5 min; the baseline heart rate and blood pressure were measured before the test. Then, they were asked to immediately wear the VR device to watch the neutral pictures (landscape, still life) for 5 min; Then, VR-VAS and DDQ scores were immediately recorded, blood pressure and heart rate were measured then they were allowed to rest for 5 min. After cue induction, the baseline blood pressure and heart rate were measured. The VR device was then put on to watch the MA equipment and objects, as well as the pictures and videos of MA inhaling ice for 5 min with sound effects. After completion of the prompt procedure, the VR-VAS and DDQ scores were immediately recorded, and the blood pressure and heart rate were measured (the heart rate measuring instrument was the Polar meter V800 made in Finland, whereas the sphygmomanometer was Omron T10) (Figure 3).

### Biochemical Analysis

The plasma concentration of IL-1β (Reagent test kit:ml058059-2;batch number: LOT20181205A), TNF-α (Reagent test kit:ml077385-2; batch number: LOT20190207A) and IL-6 (Reagent test kit: ml027379-2;batch number: LOT20190508A) was measured using ELISA. The company name is Shanghai Enzyme Link Biotechnology Co., Ltd.

### Statistical Methods

Mixed-effects analysis of variance was used to evaluate the differences in VR-VAS scores, blood pressure, heart rate, etc. between baseline and neutral induction; baseline and cue-induced NCC group, CC group, and CE group. Repeated measures analysis of variance was used to analyze the differences in different groups and times. The effect of the interaction on VR-VAS, DDQ scores, and plasma levels was assessed among patients in MA withdrawal. In the case of *p* < 0.05, the Bonferroni method was used for post-test comparisons between groups. The SPSS 22.0 software was used to perform statistical analysis on the acquired data.

## Results

### Results of VR-VAS, Heart Rate, and Blood Pressure Before and After Induction

**Figure 4:**
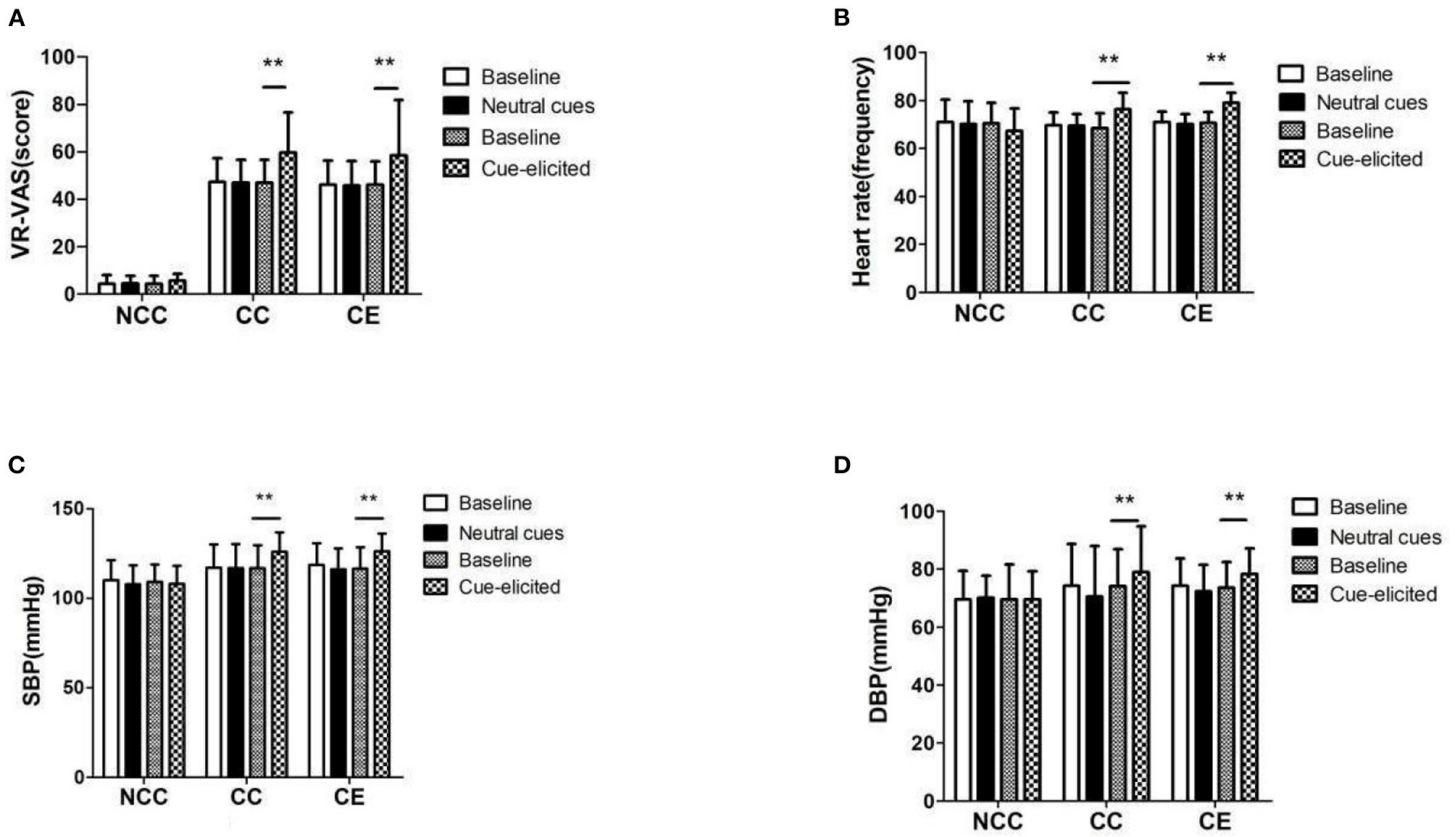
**(A–D)** VR-VAS scores, blood pressure, and heart rate measurement results before and after induction (**p < 0.01, difference between baseline and cue-elicited). Baseline, a quiet state without the influence of external factors; Neutral cues: Watching nature and landscape paintings; Cue-elicited, Watch pictures and videos related to MA; VR-VAS, Inducing cravings with cues measured by VR; Heart rate, the number of heart beats per minute; SBP, systolic blood pressure; DBP, diastolic blood pressure.

The cue induction results showed that group and time influenced the changes in VR-VAS (F(2,71) = 146.87, *p* < 0.001; F(1,71) = 22.198, *p* < 0.001), heart rate (F(2,71) = 5.96, *p* < 0.001; F(1,71) = 35.223, *p* < 0.001), systolic blood pressure (F(2,71) = 9.782, *p* < 0.001; F(1,71) = 95.314, *p* < 0.001) and diastolic blood pressure (F(2,71) = 2.849, *p*>0.05; F(1,71) = 38.357, *p* < 0.001) induced by cue. An interaction was noted between group and time (F(2,71) = 3.629, *p* < 0.05; F(2,71) = 24.438, *p* < 0.001; F(2,71) = 23.395, *p* < 0.001; F(2,71) = 8.966, *p* < 0.000). After neutral induction, the VR-VAS score, heart rate, systolic blood pressure, and diastolic blood pressure in the NCC, CE, and CC groups did not change (*p* > 0.05); after induction, the VR- VAS, heart rate, systolic and diastolic blood pressure in the NCC group did not change. VR- VAS, heart rate, systolic blood pressure and diastolic blood pressure increased in CC group (*p* > 0.05) and CC group (*p* < 0.01) (Figure 4). Each value in the figure is represented by Means ± SD.

### VR-VAS and DDQ Pre-exercise and Post-exercise Results

**Figure 5:**
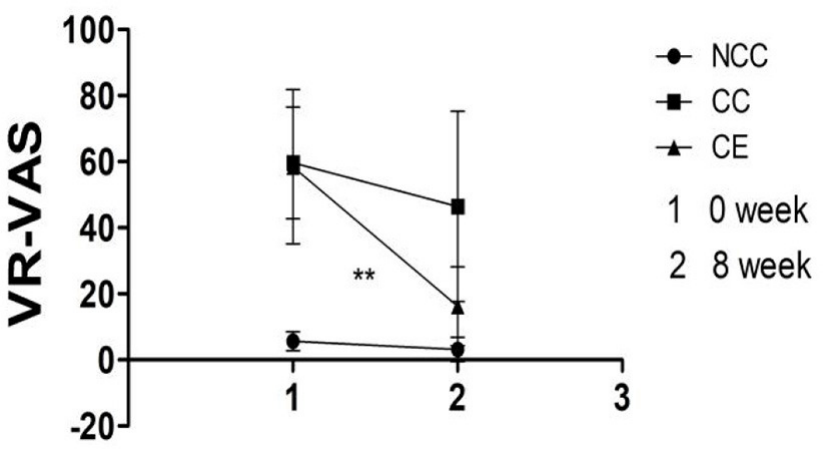
Comparison of VR-VAS scores pre-exercise and post-exercise (**p < 0.01, difference between 0 week and 8 week). 1,Week 0 is the score of VR-VAS; 2, Week 8 is the score of VR-VAS; VR-VAS, inducing cravings with cues measured by VR.

Results indicated that both group and time affected the change of VR- VAS (*p* < 0.001), with an interaction between them (*p* < 0.001). The VR-VAS of the CC and CE groups decreased after 8 weeks compared to pre-exercise (*p* < 0.01) (Figure 5). Table 2 illustrates the differences between group, time, and group^*^time.

**Table 2:** The effect of exercise on VR-VAS.

**Group**	**Pre-exercise**	**Post-exercise**	***P*** **(intergroup)**	***P*** **(time)**	***P*** **(interaction)**
NCC group	5.65 ± 2.94	3.15 ± 3.70	<0.001	<0.001	<0.001
CC group	59.63 ± 16.92ΔΔ	46.48 ± 28.78ΔΔ^**^			
CE group	58.48 ± 23.37ΔΔ	16.22 ± 11.89▴▴**			

*ΔΔp < 0.01 compared with NCC group; ▴▴p < 0.01 compared with CC group; and*

** *p < 0.01 compared with pre-exercise*.

**Figure 6:**
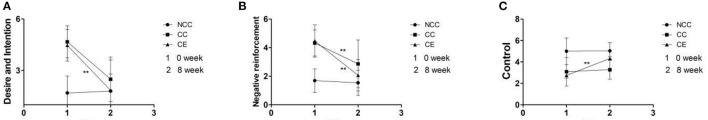
**(A–C)** Comparison of DDQ scores pre-exercise and post-exercise (**p < 0.01, difference between 0 week and 8 week). 1,Week 0 is the score of VR-VAS; 2,Week 8 is the score of VR-VAS; Desire and Intention, desire for MA; Negative reinforcement, repeated use of MA to relieve withdrawal symptoms; Control, the ability to control MA.

The obtained results indicate that both group and time affected changes in the three dimensions (*p* < 0.01), with an interaction between them (*p* < 0.001). The “desire and intention” and “negative reinforcement” of the CC group and the CE group post-exercise were significantly reduced compared to pre-exercise (*p* < 0.01); however, the CE group significantly decreased. At the same time, the “control” of the CE group significantly increased post-exercise (*p* < 0.01), however, there was no change in the CC group (Figure 6). Table 3 details the difference between the group, time, group^*^time.

**Table 3:** The effect of exercise on DDQ.

**Dimension**	**Group**	**Pre-exercise**	**Post-exercise**	***P*** **(intergroup)**	***P*** **(time)**	***P*** **(interaction)**
Desire and intention	NCC group	1.69 ± 0.98	1.80 ± 1.81	<0.001	<0.001	<0.001
	CC group	4.67 ± 0.93ΔΔ	2.49 ± 1.30ΔΔ			
	CE group	4.47 ± 0.92ΔΔ	1.84 ± 0.95ΔΔ ▴▴^**^			
Negative reinforcement	NCC group	1.69 ± 0.82	1.54 ± 0.88	<0.001	<0.001	<0.001
	CC group	4.33 ± 0.90ΔΔ	2.87 ± 1.67ΔΔ ^**^			
	CE group	4.47 ± 1.12ΔΔ	2.07 ± 1.10ΔΔ ▴▴^**^			
Control	NCC group	5.00 ± 1.24	5.03 ± 0.77	<0.001	<0.01	<0.01
	CC group	3.09 ± 13.4ΔΔ	3.28 ± 0.89ΔΔ			
	CE group	2.74 ± 0.25ΔΔ	4.32 ± 0.17ΔΔ ▴▴^**^			

*ΔΔp < 0.01 compared with NCC group; ▴▴p < 0.01 compared with CC group; and*

** *p < 0.01 compared with pre-exercise*.

### Measurement Results of Plasma IL-1β, TNF-α, and IL-6 Concentration Pre-exercise and Post-exercise

**Figure 7:**
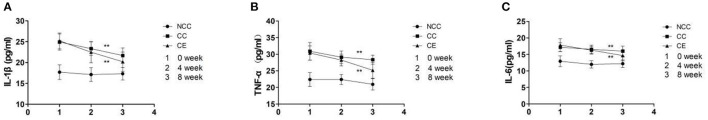
**(A–C)** The effect of exercise on plasma IL-1β, TNF-α, IL-6 concentration (**p < 0.01, difference between 4 week and 8 week). 1, Plasma IL-1β, TNF-α, IL-6 concentration at week 0; 2, Plasma IL-1β, TNF-α, IL-6 concentration at week 4; 3, Plasma IL-1β, TNF-α, IL-6 concentration at week 8.

Results showed that the concentration of IL-1 β, TNF - α, and IL-6 were influenced by group and time (*p* < 0.001), with an interaction between them (*p* < 0.001). A comparison of week zero and week eight indicated that the plasma concentrations of IL-1β, TNF - α, and IL-6 in the CC group and CE group were significantly lower than those in the CC group (*p* < 0.01), however, there was a remarkable decrease in the CE group (Figure 7). Table 4 shows the difference between the group, time, group^*^time.

**Table 4:** The effect of exercise on plasma IL-1β, TNF - α, and IL-6 concentrations.

**Dimension**	**Group**	**0 week**	**4-weeks**	**8-weeks**	***P*** **(inter-group)**	***P*** **(time)**	***P*** **(inter-action)**
IL-1β (pg/ml)	NCC group	17.69 ± 1.76	17.13 ± 1.66	17.32 ± 1.51	<0.001	<0.001	<0.001
	CC group	24.93 ± 1.94 ΔΔ	23.34 ± 1.61 ΔΔ	21.69 ± 1.82 ΔΔ^**^			
	CE group	25.20 ± 1.90 ΔΔ	22.49 ± 2.53 ΔΔ	20.20 ± 2.31 ΔΔ▴▴^**^			
TNF-α (pg/ml)	NCC group	22.38 ± 2.07	22.39 ± 1.51	20.93 ± 1.71	<0.001	<0.001	<0.001
	CC group	30.88 ± 2.61 ΔΔ	29.10 ± 1.89 ΔΔ^**^	28.37 ± 1.33 ΔΔ^**^			
	CE group	30.34 ± 2.13 ΔΔ	28.24 ± 1.79 ΔΔ▴	25.17 ± 2.45 ΔΔ▴▴^**^			
IL-6 (pg/ml)	NCC group	12.93 ± 1.62	11.98 ± 1.12	12.20 ± 1.12	<0.001	<0.001	<0.001
	CC group	17.18 ± 1.31 ΔΔ	16.54 ± 1.27 ΔΔ	16.00 ± 1.52 ΔΔ^**^			
	CE group	17.85 ± 1.94 ΔΔ	16.29 ± 1.19 ΔΔ	14.66 ± 1.61 ΔΔ▴▴**			

*ΔΔp < 0.01 compared with NCC group; ▴p < 0.05 compared with CC group; ▴▴p < 0.01 compared with CC group; and*

** *p < 0.01 compared with week zero*.

## Discussion

### The Effect of Aerobic Combined Resistance Training on the Levels of Plasma IL-1β, TNF-α, and IL-6

One previous study reported that exercise protects an individual against the MA-induced systemic increase of pro-inflammatory cytokines levels. Additionally, exercise reduces the extent of BBB destruction and improves related microenvironmental changes to reduce MA-induced neurotoxicity (37). This work found that 8 weeks of aerobic combined resistance training significantly reduces the levels of IL-1β, TNF-α, and IL-6 in peripheral blood. After 8 weeks, there was a slight decrease in the plasma level of IL-1β in the CC group, whereas the CE group had a remarkable decrease. Considering that the degree of craving was not different, exercise had superior effects to conventional nursing in rehabilitation centers. Besides, the plasma TNF-α levels of the CC and CE groups showed significant differences in the 8th week. In contrast, the plasma TNF-α levels of the CE group were significantly decreased. This confirms the apparent effect of exercise on the improvement of plasma TNF-α levels. The plasma IL-6 levels of the CC and CE groups decreased after 8 weeks, however, the CE group showed more changes than the CC group. This indicates that aerobic combined resistance training has a significant anti-inflammatory effect on individuals in MA withdrawal. The slight decrease in inflammation levels in the CC group may be attributed to the influence of environmental regulation and adjustment of self-cognition in the rehabilitation center. This is an interesting finding. We found that the level of inflammation in the peripheral system can be improved.

### The Effect of Aerobic Combined Resistance Training on the Degree of Cue-Induced Craving Under VR Exposure

Cue-induced cravings under VR exposure effectively reflect the degree of addiction under laboratory conditions (14, 15, 38). DDQ was used as a supplementary scale to supplement VR-VAS evaluation; its accuracy was increased by incorporating correlation analysis results of the two scales (39). Of note, cravings caused by cues are related to an increase in blood pressure, heart rate, and changes in skin temperature (40). This theory is consistent with psychobiological activation. The group pattern of change in craving from pre-post exercise (Figure 4A) paralleled the group pattern of change in Heart Rate (Figure 4B): In both cases the pattern of scores was as follows: Neutral = Baseline and Cue-Elicited > Baseline, and the cue effect was parallel for both outcome measures (craving and heart rate) and equivalent in Group CC and in Group CE, with no corresponding change as a function of cues in Group NCC. Therefore the cue manipulation was equally effective in MA patients who did vs. those who did not receive the exercise intervention. The parallel pattern for selfreported craving and heart rate not only demonstrates that cue exposure significantly altered physiological function, but also provides a procedural check on the manipulation because physiological reactivity to a cue is much less susceptible to distortion than self-report. This also explains the principle of psychobiological activation and to some extent the rationality of grouping. Nonetheless, screening personnel in the NCC group was problematic because the rehabilitation center was isolated from the external environment, thus decreasing accessibility and cravings. This may be because the reason for their drug use is the effect of the surrounding environment, but they cannot get rid of the drug use environment. Nonetheless, the rehabilitation center mediates the isolation and insulation at this time. Thus, after a period of environmental isolation in the NCC group, a smaller heart rate and blood pressure response will occur when the cue is exposed; this is also related to the sensitivity of an individual to the cue exposure. Considering that addiction mental scales are different from objective indicators including blood, repeated measurements are taken within a short period to improve the sensitivity and accuracy of the true results. As such, the psychological scales were only collected at two measurement points, at week zero and week eight. The findings indicated a certain “ceiling effect” between baseline and cue-induced cravings in the pre-test; the VR-VAS score of the NCC group also had a “floor effect” after cue-induced exposure. However, a consistently high craving score precisely reflects the “true” high level (37). Based on the severity of cravings in the pre-test, the CC and CE groups also showed higher levels of cravings at baseline and after cue-induced exposure.

In recent years, while the good effects of physical exercise have been recognized by most people, it has been slowly applied to the field of drug addiction treatment. Short-term aerobic exercise improves the craving of MA-dependent people and increases the inhibition capacity (5, 19, 21); it has a remarkable effect on improving the emotional state of MA-dependent people (26). Aerobic combined resistance training enhances the physical health of MA withdrawal (20, 23) as well as the degree of anxiety and depression (22). In animal experiments, exercise reduces the intake and seeking of MA, and in turn, reduces neurotoxicity caused by MA (41). Our research shows that aerobic combined resistance training significantly reduces the cue-induced cravings of MA withdrawal patients.

### Relationship Between Plasma IL-1β, TNF-α, and IL-6 Levels and the Degree of Cue-Induced Craving

Chronic MA exposure increases BBB permeability and hippocampal IL-1β levels (37). Also, IL-1β levels significantly increased in the analysis of the sample (37) MA abuse increases the level of TNF-α in the central nervous system, and the nucleus accumbens; this is closely related to drug dependence, highly expresses TNF-α mRNA and protein (42). Previous studies reported that deleting the TNF-α gene influences addictive behaviors in wild-type mice, including automatic MA seeking, motivation to MA, prompting-induced cravings, and drug-seeking behaviors (3). IL-6 promotes neuroinflammatory response (43) and brain pathological changes [45], entering the functional areas of the brain related to various diseases. This indicates that peripheral inflammatory factors are linked to central nervous system damage and drug-seeking.

We revealed similar plasma inflammation levels in the CC and CE groups. Interestingly, the plasma inflammation level is different when the craving degree is significantly different between the NCC and CC groups. This indicates that plasma inflammation levels among patients in MA withdrawal are significantly related to drug cravings caused by brain inflammation. This may corroborate with results of previous studies on the damage of peripheral and central nervous system structures a well as mental disorders.

## Conclusion

This study confirmed that 8 weeks of incremental load aerobic combined with resistance training reduces peripheral inflammation and significantly reduces the level of craving for MA.

## Deficiencies and Future Prospects

The main purpose of this study was to explore the relationship between plasma IL-1β, TNF- α and IL-6 levels and cue-induced cravings. We also studied whether 8 weeks of aerobic combined resistance training can reduce the levels of IL-1 β, TNF – α, and IL- 6 during MA extraction, and improve the cravings induced by cue. This will lay the foundation for further research on whether inflammatory factors such as IL-1 β, TNF – α, and IL-6 can be used as plasma markers of craving during the withdrawal patients period. However, this study has some limitations. Although the effect of exercise intervention for 8 weeks is similar to that in previous studies, the 8-week exercise intervention time is relatively short and cannot predict the subtle changes of various indicators after a long time. At the same time, this article only conducted research on men, and did not explore the differences between men and women. Although VR-VAS and DDQ combined with objective indicators can accurately reflect the physiological response in the induction process, the number of objective indicators is small. The experimental group in future studies should increase skin resistance, eye movement, near-infrared and other tests to enhance the examples of objective indicators. The studies should also use FMRI to analyze the neuro-toxic inflammatory injury in the reward area of the central nervous system, and then observe the improvement effect of craving degree. In addition, they should use multiple indicators and multiple dimensions to explore the correlation between the degree of inflammation and craving of MA during long-term withdrawal patients, and verify whether exercise can improve the effect.

## Data Availability Statement

The original contributions presented in the study are included in the article/supplementary files, further inquiries can be directed to the corresponding author/s.

## Ethics Statement

The studies involving human participants were reviewed and approved by the Ethics Committee of Hunan Normal University approved this study (Batch number : 16-2010). The patients/participants provided their written informed consent to participate in this study.

## Author Contributions

JW, JZ, LZ, and CL conceived and designed the experiments. JW screened experimental subjects, signed the informed consent process, and conducted the exercise intervention. All authors contributed to the article and approved the submitted version.

## Funding

This research is supported by the National Key Research and Development Program (2016YFC0800908), research and innovation program for Postgraduates of Physical Education College of Hunan Normal University (TYCX2019B007), and the Scientific Research Project of Hunan Provincial Department of Education (19C1133).

## Conflict of Interest

The authors declare that the research was conducted in the absence of any commercial or financial relationships that could be construed as a potential conflict of interest.

## Publisher's Note

All claims expressed in this article are solely those of the authors and do not necessarily represent those of their affiliated organizations, or those of the publisher, the editors and the reviewers. Any product that may be evaluated in this article, or claim that may be made by its manufacturer, is not guaranteed or endorsed by the publisher.
